# Supplementary material for: Mice lacking triglyceride synthesis enzymes in adipose tissue are resistant to diet-induced obesity
Source: eLife. 2023 Oct 2;12:RP88049. doi: 10.7554/eLife.88049 (PMC10545428; doi:10.7554/eLife.88049)
Supplement: Supplementary file 1. [file elife-88049-supp1.pdf]

Supplementary File 1

Primers used for quantitative real-time PCR analysis of mouse genes		
Dgat1	Fwd:<br>Rev:	GGAATATCCCCGTGCACAA<br>CATTTGCTGCTGCCATGTC
Dgat2	Fwd:<br>Rev:	CCGCAAAGGCTTTGTGAA<br>GGAATAAGTGGGAACCAGATCAG
Srebp1c	Fwd:<br>Rev:	GGAGCCATGGATTGCACATT<br>GGCCCGGGAAGTCACTGT
Acc	Fwd:<br>Rev:	GATGAACCATCTCCGTTGGC<br>GACCCAATTATGAATCGGGAGTG
Fas	Fwd:<br>Rev:	GGAGGTGGTGATAGCCGGTAT<br>TGGGTAATCCATAGAGCCCAG
Scd1	Fwd:<br>Rev:	TTCTTGCGATACACTCTGGTGC<br>CGGGATTGAATGTTCTTGTCGT
Ucp1	Fwd:<br>Rev:	AGGCTTCCAGTACCATTAGGT<br>CTGAGTGAGGCAAAGCTGATTT
CideA	Fwd:<br>Rev:	TGCTCTTCTGTATCGCCCAGT<br>GCCGTGTTAAGGAATCTGCTG
Pgc1α	Fwd:<br>Rev:	TTCATCTGAGTATGGAGTCGCT<br>GGGGGTGAAACCACTTTTGTAA
Ppar1α	Fwd:<br>Rev:	AATGCAATTCGCTTTGGAAG<br>GGCCTTGACCTTGTTCATGT
Cpt1α	Fwd:<br>Rev:	GAACCCCAACATCCCCAAAC<br>TCCTGGCATTGTCCTGGAAT
Mcad	Fwd:<br>Rev:	AGGTTTCAAGATCGCAATGG<br>CTCCTTGGTGCTCCACTAGC
Lcad	Fwd:<br>Rev:	TCCATGGCAAAATACTGGGC<br>TTGCAATCGGGTACTCCCAC
Xbp1s	Fwd:<br>Rev:	GGTCTGCTGAGTCCGCAGCAGG<br>AGGCTTGGTGTATACATGG
Chop	Fwd:<br>Rev:	CCACCACACCTGAAAGCAGAA<br>AGGTGAAAGGCAGGGACTCA
Bip	Fwd:<br>Rev:	ACTTGGGGACCACCTATTCCT<br>ATCGCCAATCAGACGCTCC
Atf3	Fwd:<br>Rev:	GAGGATTTTGCTAACCTGACACC<br>TTGACGGTAACTGACTCCAGC
Atf4	Fwd:<br>Rev:	CCTTCGACCAGTCGGGTTTG<br>CTGTCCCGGAAAAGGCATCC
Tnfα	Fwd:<br>Rev:	CCCTCACACTCAGATCATCTTCT<br>GCTACGACGTGGGCTACAG
F4/80	Fwd:<br>Rev:	TGACTCACCTTGTGGTCCTAA<br>CTTCCCAGAATCCAGTCTTTCC
Fgf21	Fwd:<br>Rev:	GTGTCAAAGCCTCTAGGTTTCTT<br>GGTACACATTGTAACCGTCCTC
Cyclophilin	Fwd:<br>Rev:	GGAGATGGCACAGGAGGAAA<br>CCGTAGTGCTTCAGTTTGAAGTTCT
Fwd, forward; Rev, reverse.		
