# Supplementary material for: Mice lacking triglyceride synthesis enzymes in adipose tissue are resistant to diet-induced obesity
Source: eLife. 2023 Oct 2;12:RP88049. doi: 10.7554/eLife.88049 (PMC10545428; doi:10.7554/eLife.88049)
Supplement: Source data 1. [file elife-88049-data1.zip › Source data/Figure 1-figure supplement 1-source data 1/Figure 1-figure supplement 1-source data 1.pptx]

## Slide 1
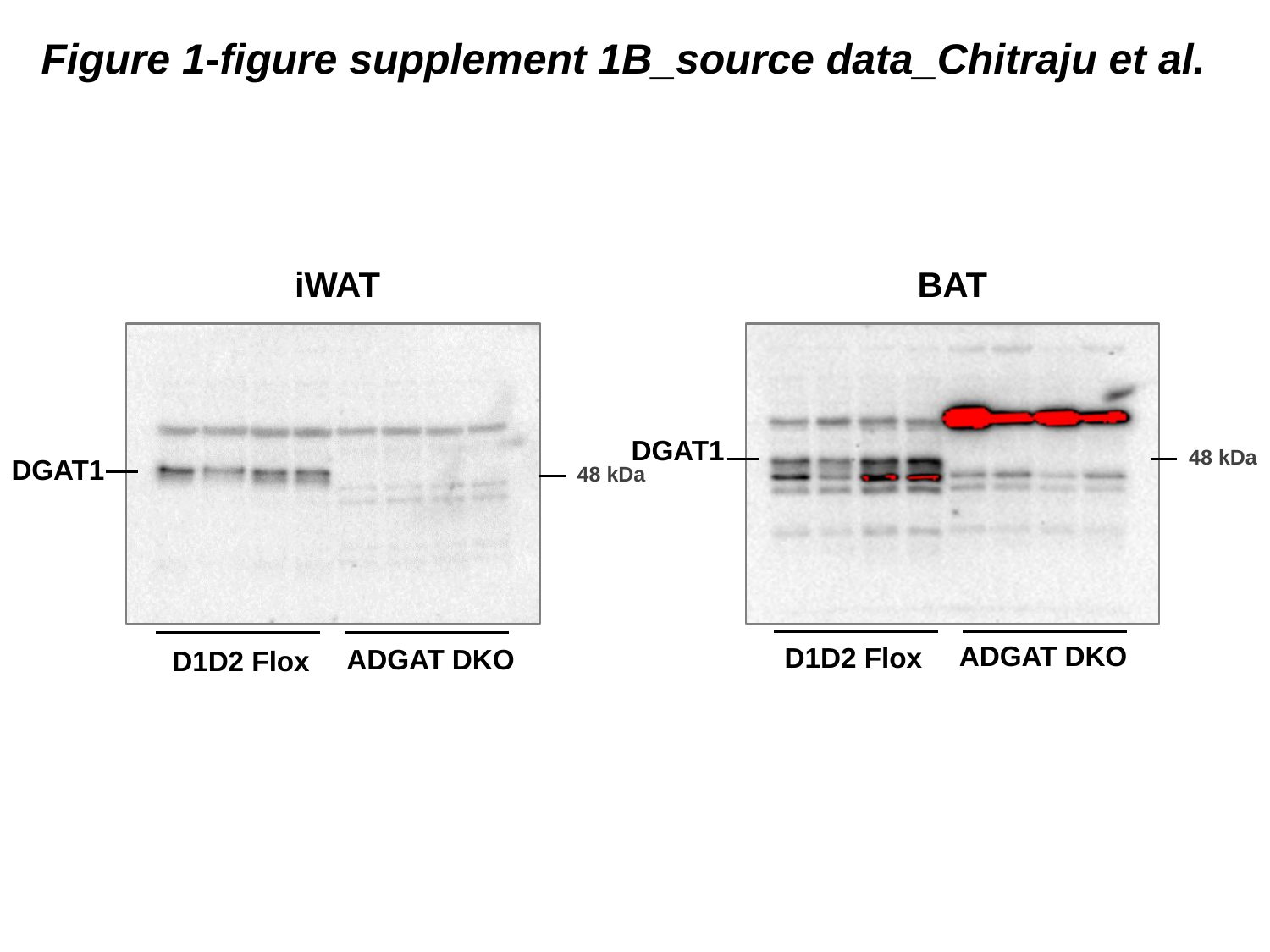

Figure 1-figure supplement 1B_source data_Chitraju et al.
iWAT
BAT
DGAT1
48 kDa
DGAT1
48 kDa
ADGAT DKO
D1D2 Flox
ADGAT DKO
D1D2 Flox

## Slide 2
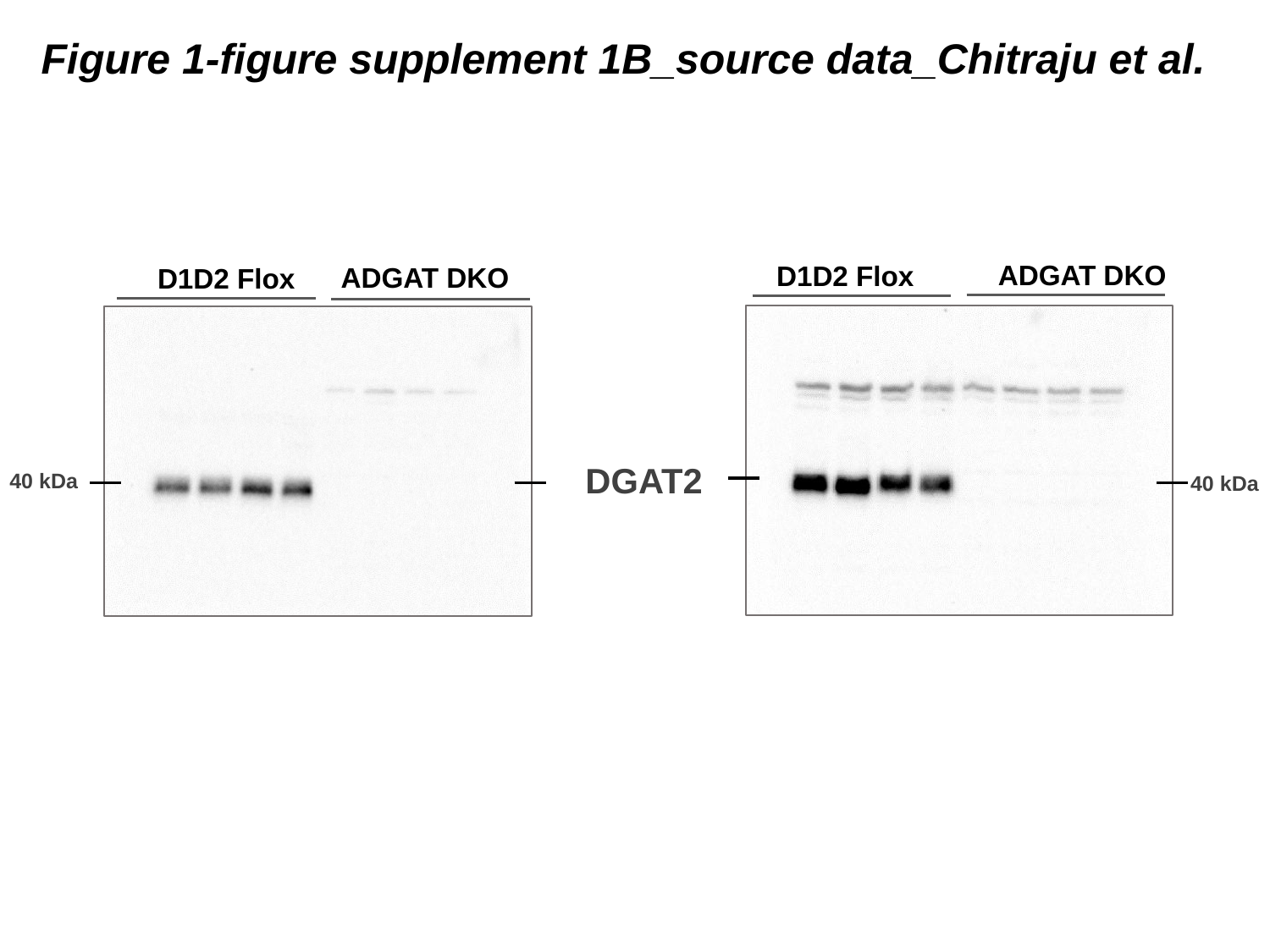

Figure 1-figure supplement 1B_source data_Chitraju et al.
ADGAT DKO
D1D2 Flox
ADGAT DKO
D1D2 Flox
DGAT2
40 kDa
40 kDa

## Slide 3
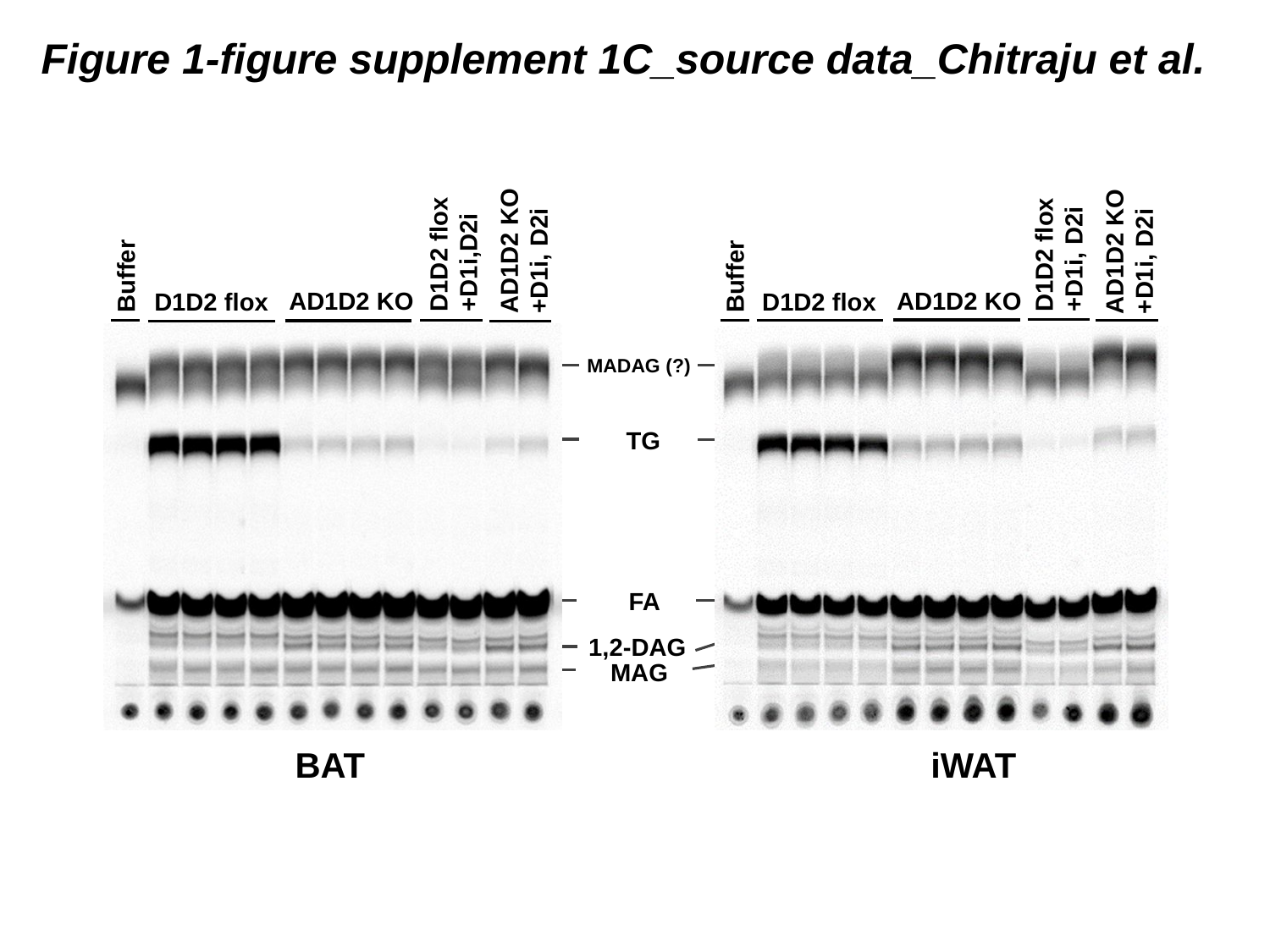

Figure 1-figure supplement 1C_source data_Chitraju et al.
AD1D2 KO
+D1i, D2i
AD1D2 KO
+D1i, D2i
D1D2 flox
+D1i,D2i
D1D2 flox
+D1i, D2i
Buffer
Buffer
AD1D2 KO
AD1D2 KO
D1D2 flox
D1D2 flox
TG
FA
1,2-DAG
MAG
BAT
iWAT
MADAG (?)

## Slide 4
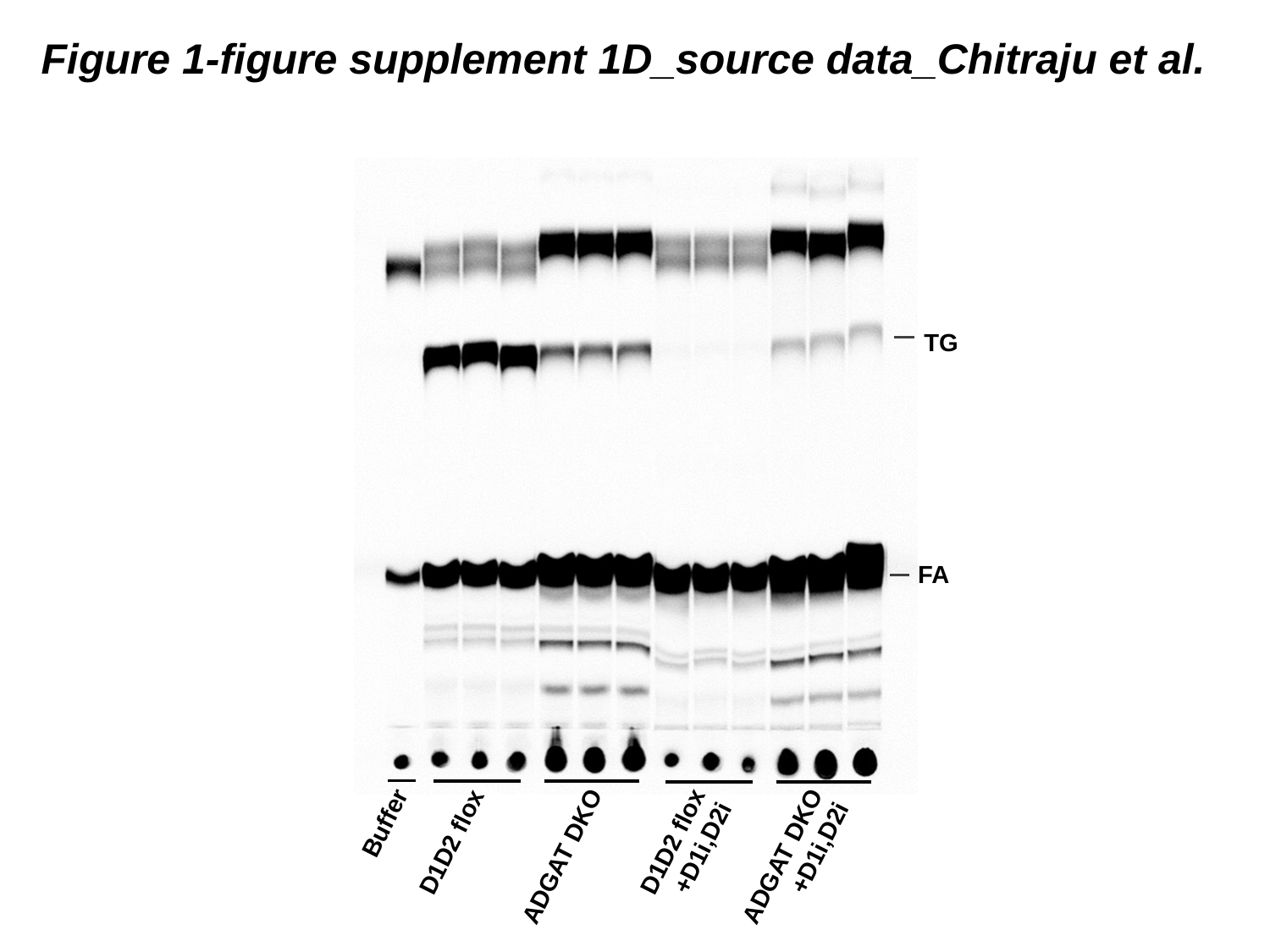

Figure 1-figure supplement 1D_source data_Chitraju et al.
Buffer
D1D2 flox
+D1i,D2i
D1D2 flox
ADGAT DKO
+D1i,D2i
ADGAT DKO
TG
FA
