# Supplementary material for: Mice lacking triglyceride synthesis enzymes in adipose tissue are resistant to diet-induced obesity
Source: eLife. 2023 Oct 2;12:RP88049. doi: 10.7554/eLife.88049 (PMC10545428; doi:10.7554/eLife.88049)
Supplement: Source data 1. [file elife-88049-data1.zip › Source data/Figure 1-Source data 2/Figure 1-Source data 2 (1).pptx]

## Slide 1
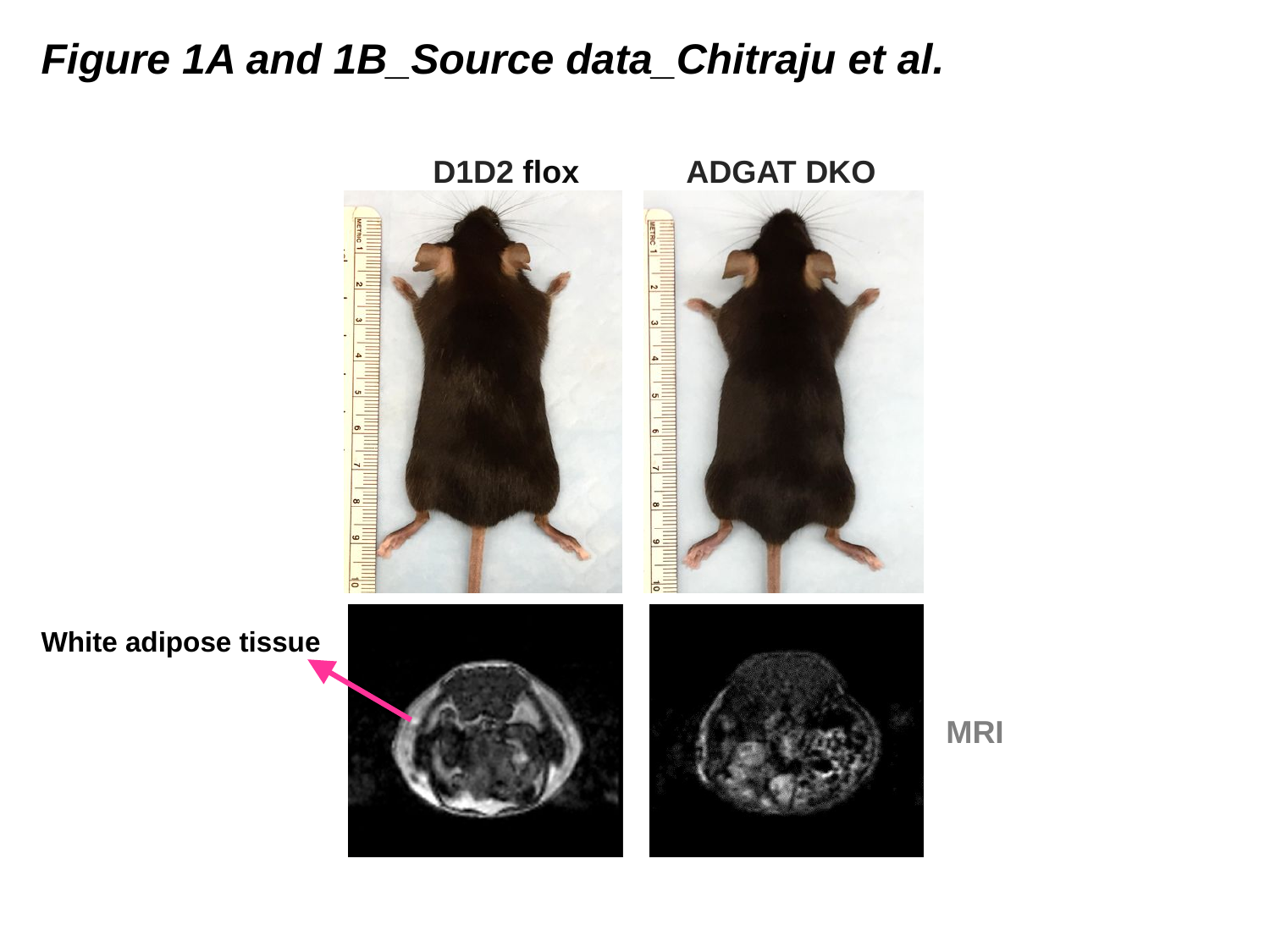

Figure 1A and 1B_Source data_Chitraju et al.
D1D2 flox
ADGAT DKO
White adipose tissue
MRI

## Slide 2
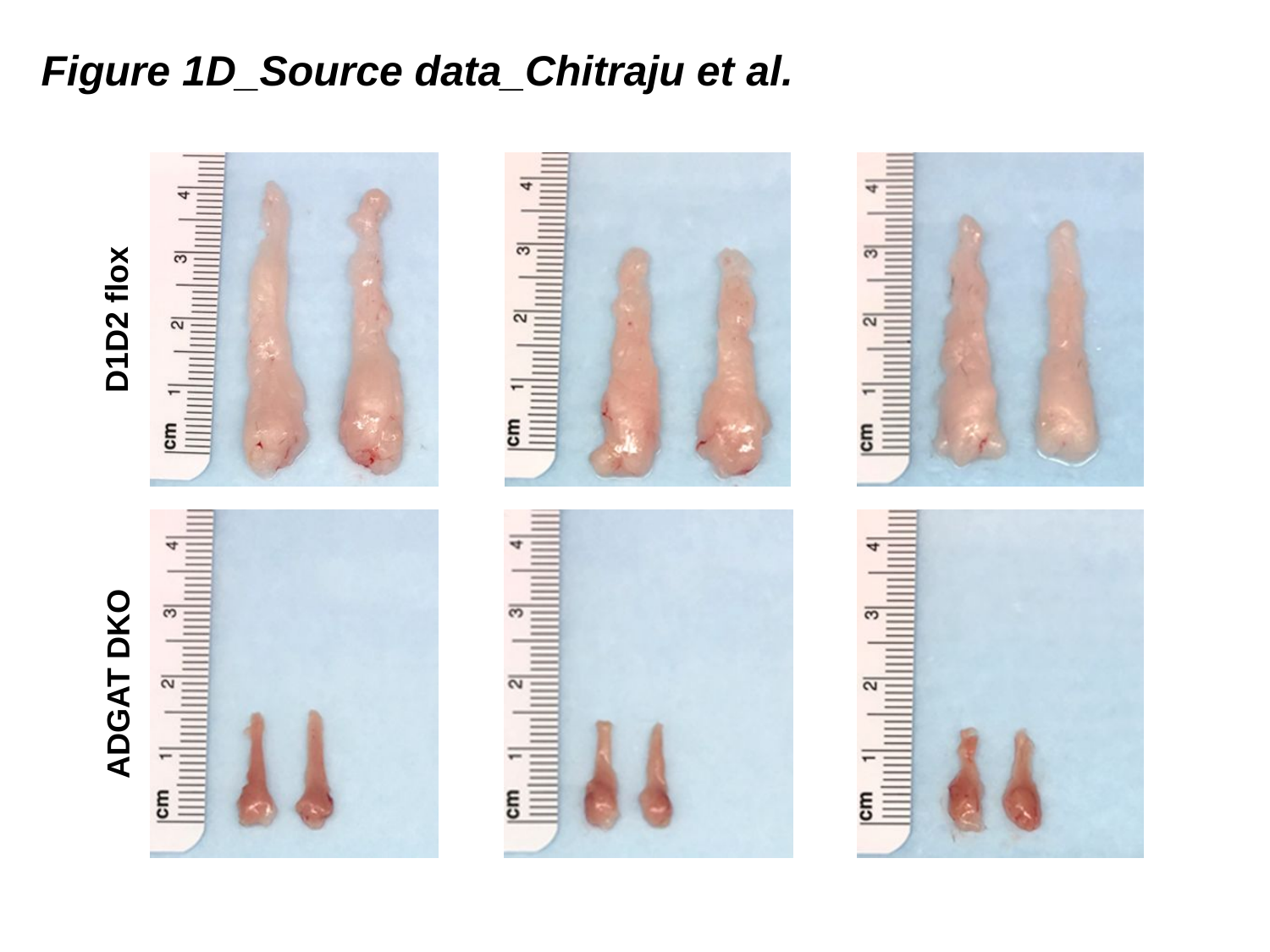

Figure 1D_Source data_Chitraju et al.
D1D2 flox
ADGAT DKO

## Slide 3
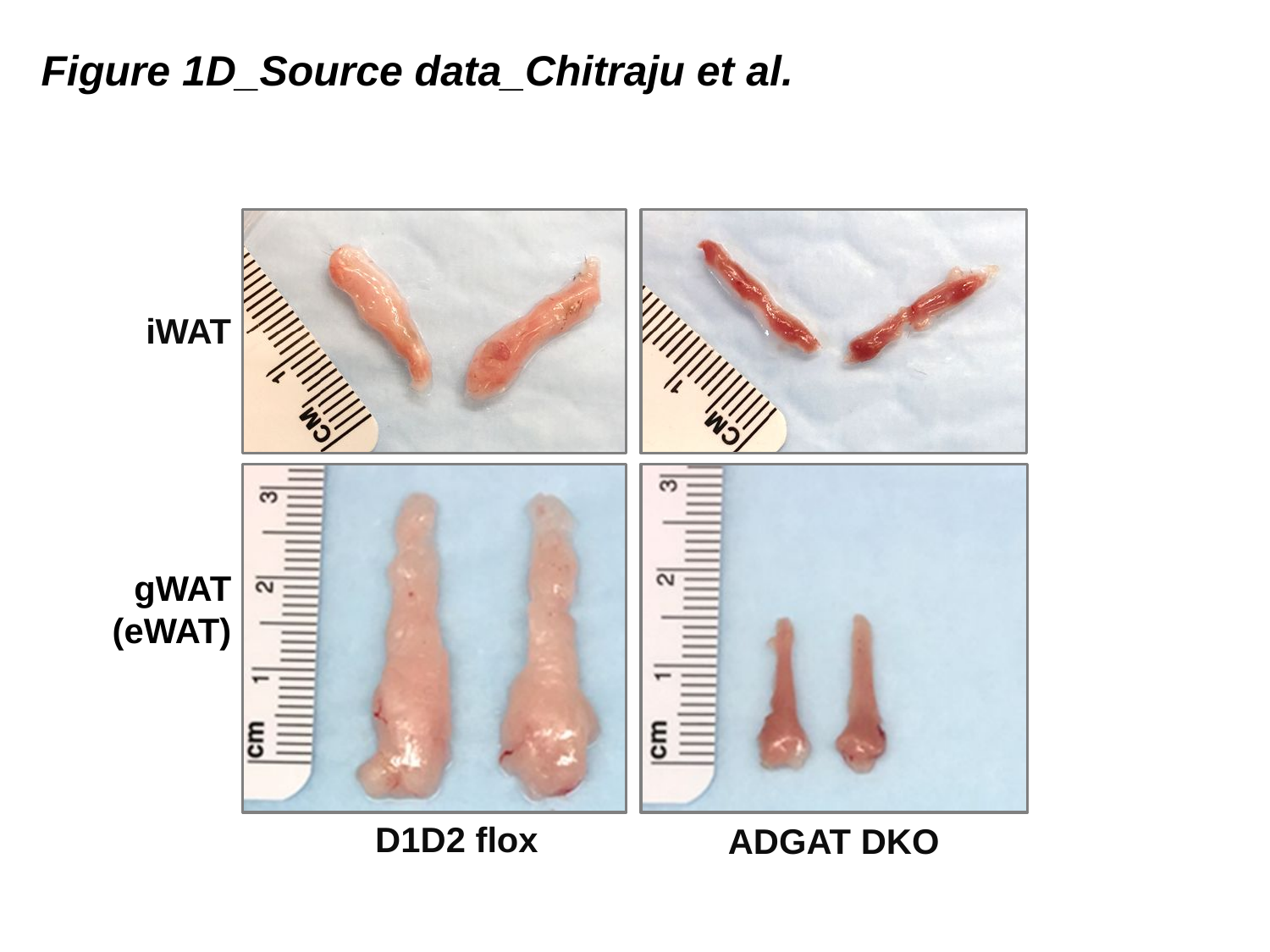

Figure 1D_Source data_Chitraju et al.
D1D2 flox
ADGAT DKO
iWAT
gWAT
(eWAT)

## Slide 4
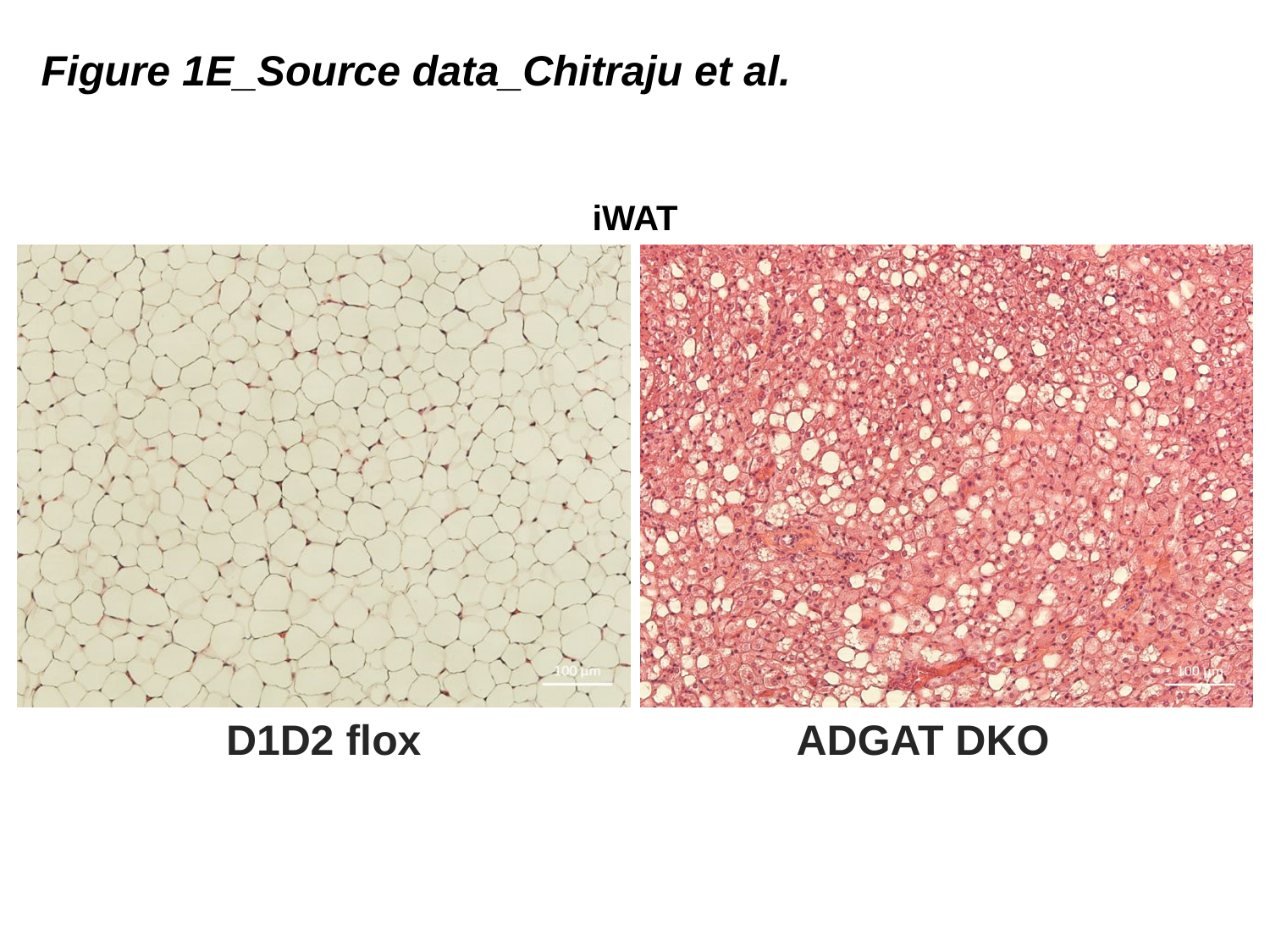

Figure 1E_Source data_Chitraju et al.
iWAT
D1D2 flox
ADGAT DKO

## Slide 5
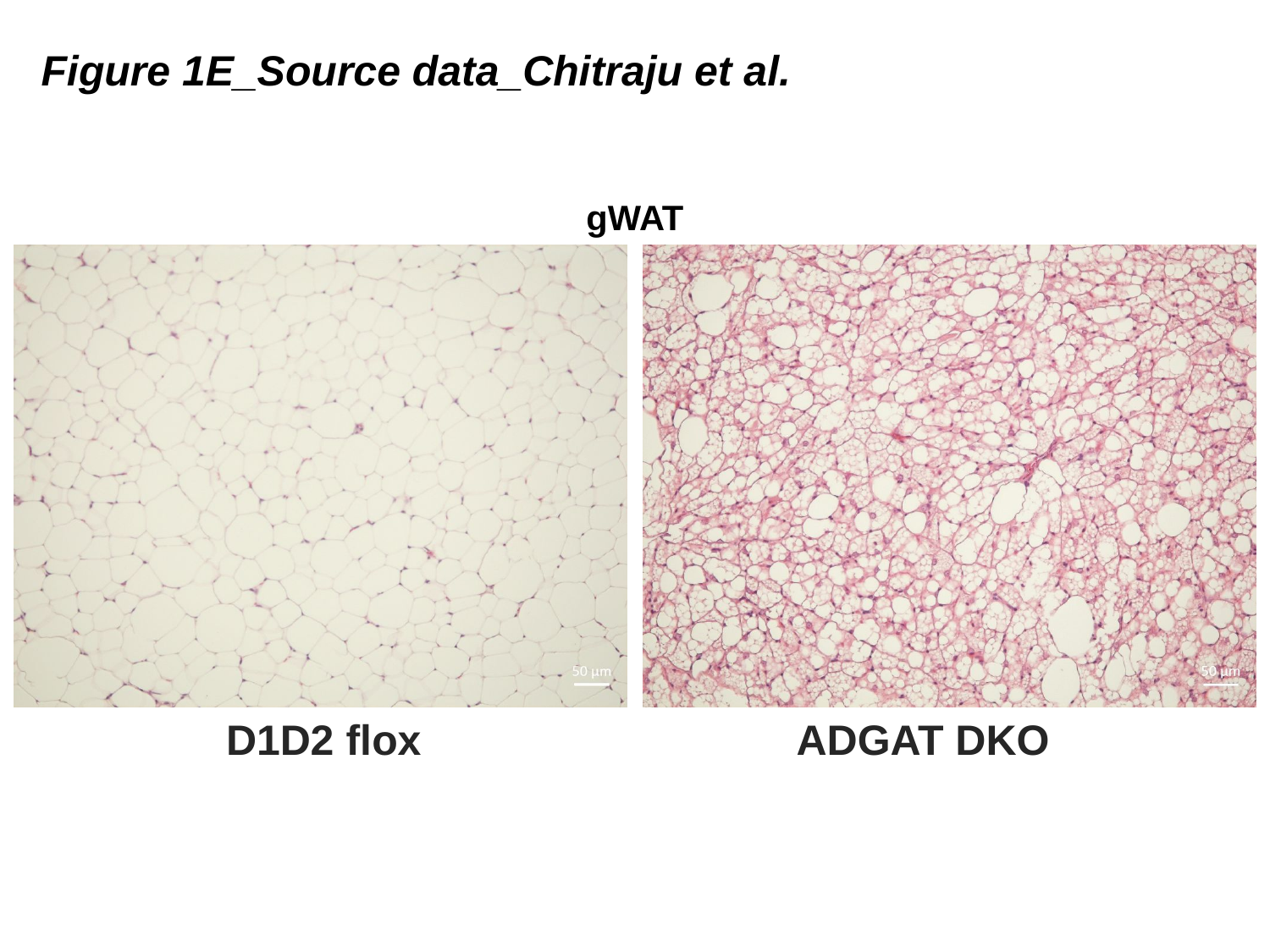

Figure 1E_Source data_Chitraju et al.
gWAT
D1D2 flox
ADGAT DKO

## Slide 6
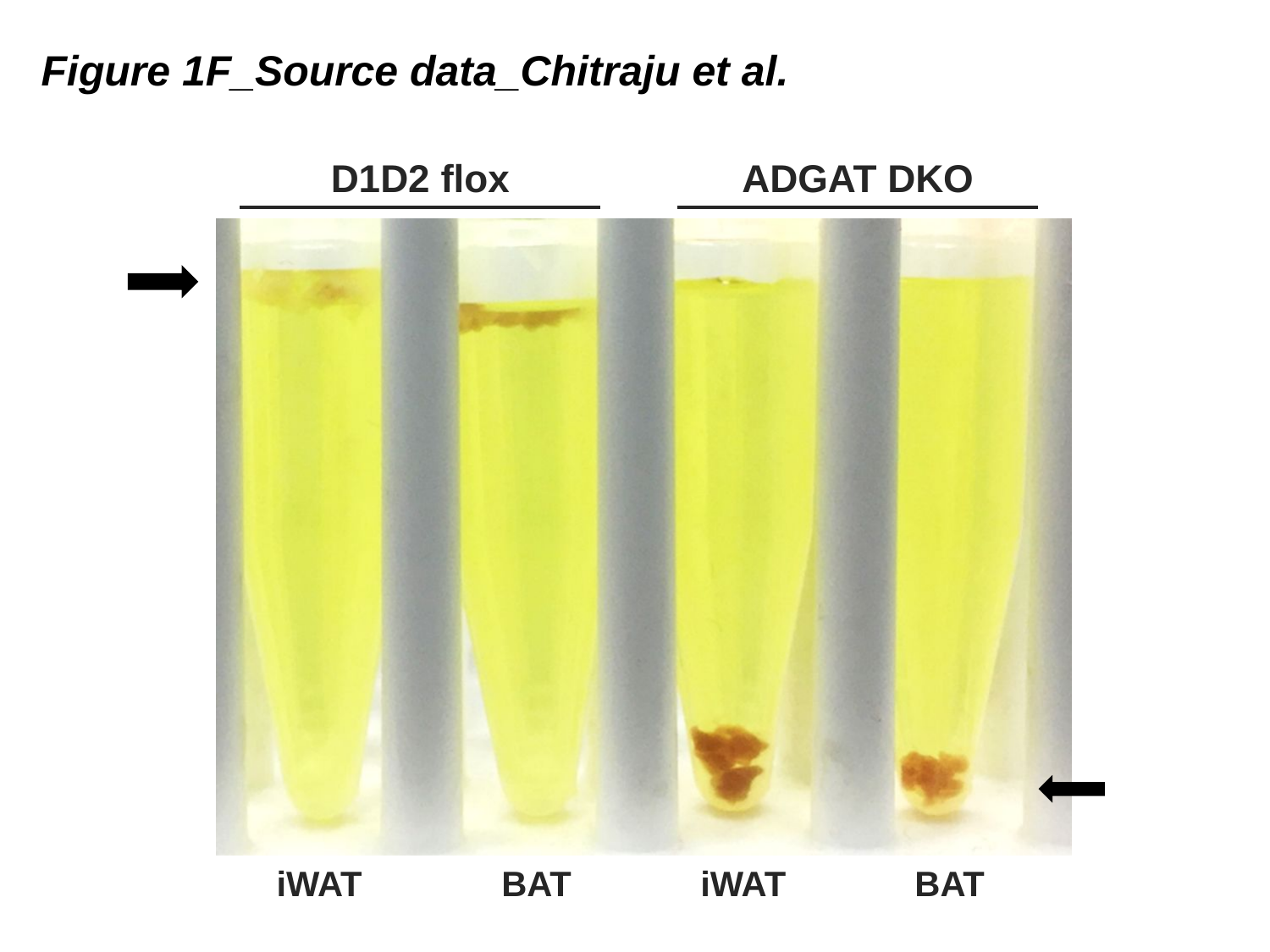

Figure 1F_Source data_Chitraju et al.
D1D2 flox
ADGAT DKO
iWAT
BAT
iWAT
BAT

## Slide 7
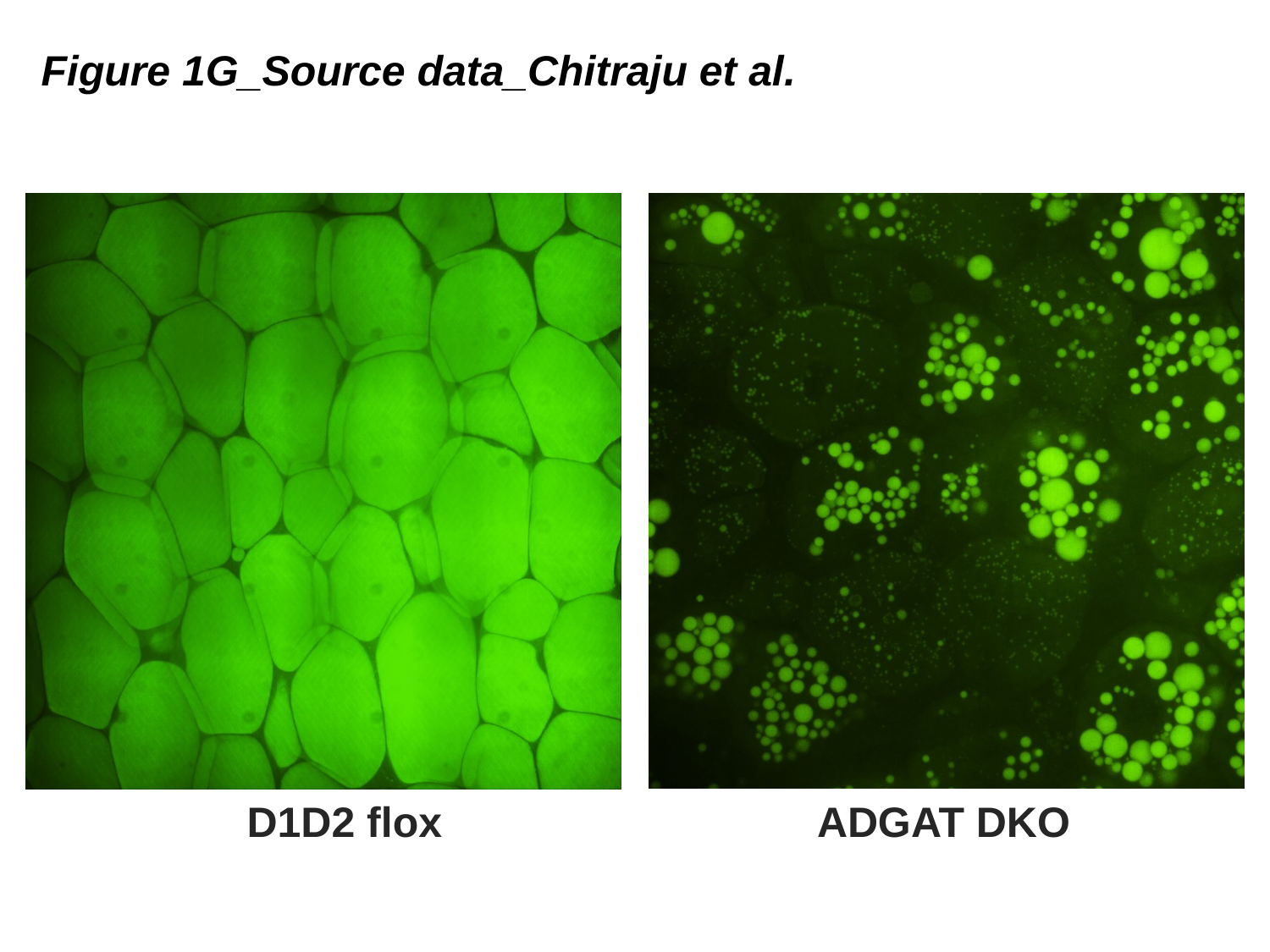

Figure 1G_Source data_Chitraju et al.
D1D2 flox
ADGAT DKO

## Slide 8
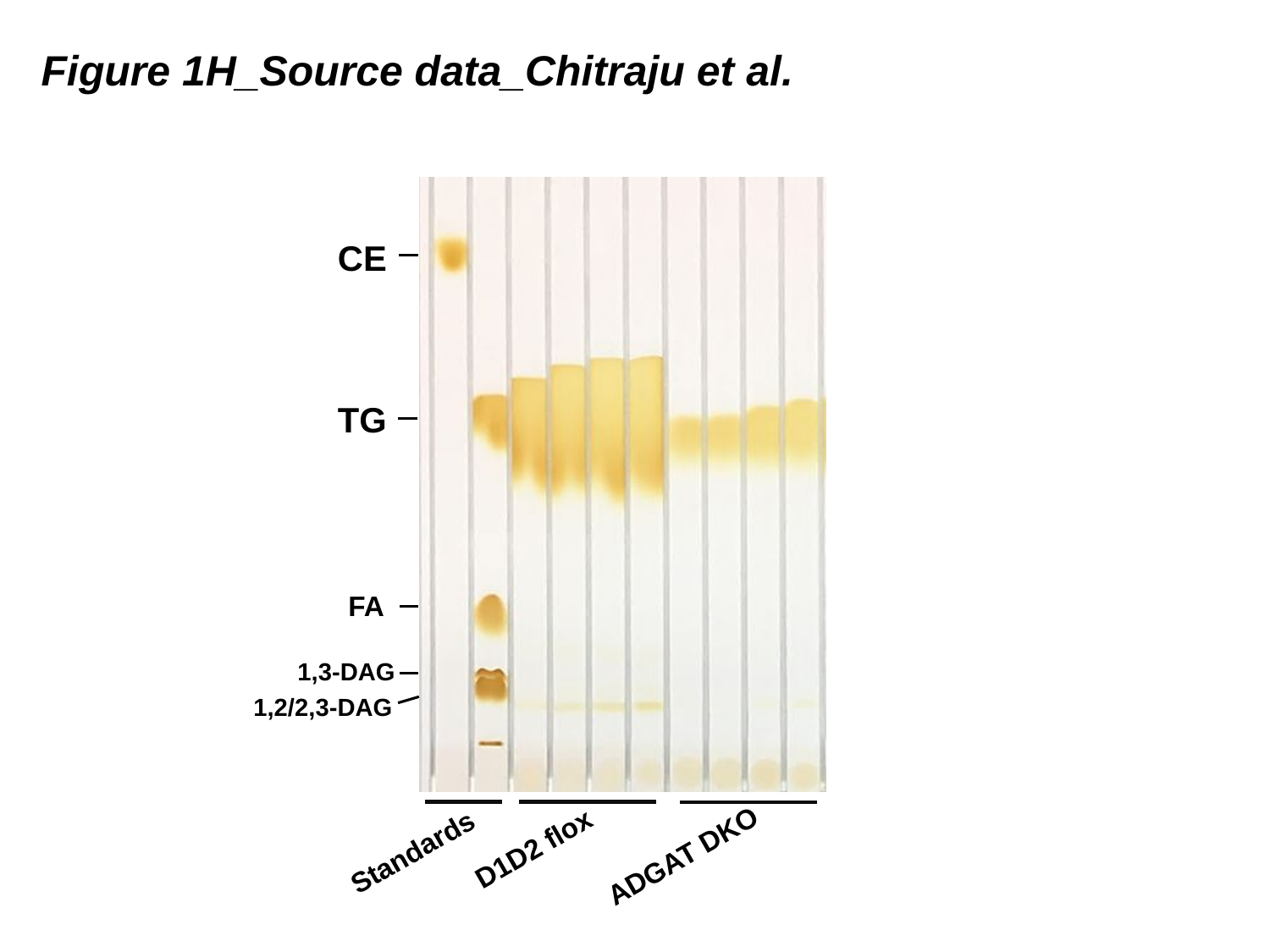

Figure 1H_Source data_Chitraju et al.
CE
TG
FA
1,3-DAG
1,2/2,3-DAG
D1D2 flox
Standards
ADGAT DKO
