# Supplementary material for: Mice lacking triglyceride synthesis enzymes in adipose tissue are resistant to diet-induced obesity
Source: eLife. 2023 Oct 2;12:RP88049. doi: 10.7554/eLife.88049 (PMC10545428; doi:10.7554/eLife.88049)
Supplement: Source data 1. [file elife-88049-data1.zip › Source data/Figure 5-source data 1/Figure 5-source data 1.pptx]

## Slide 1
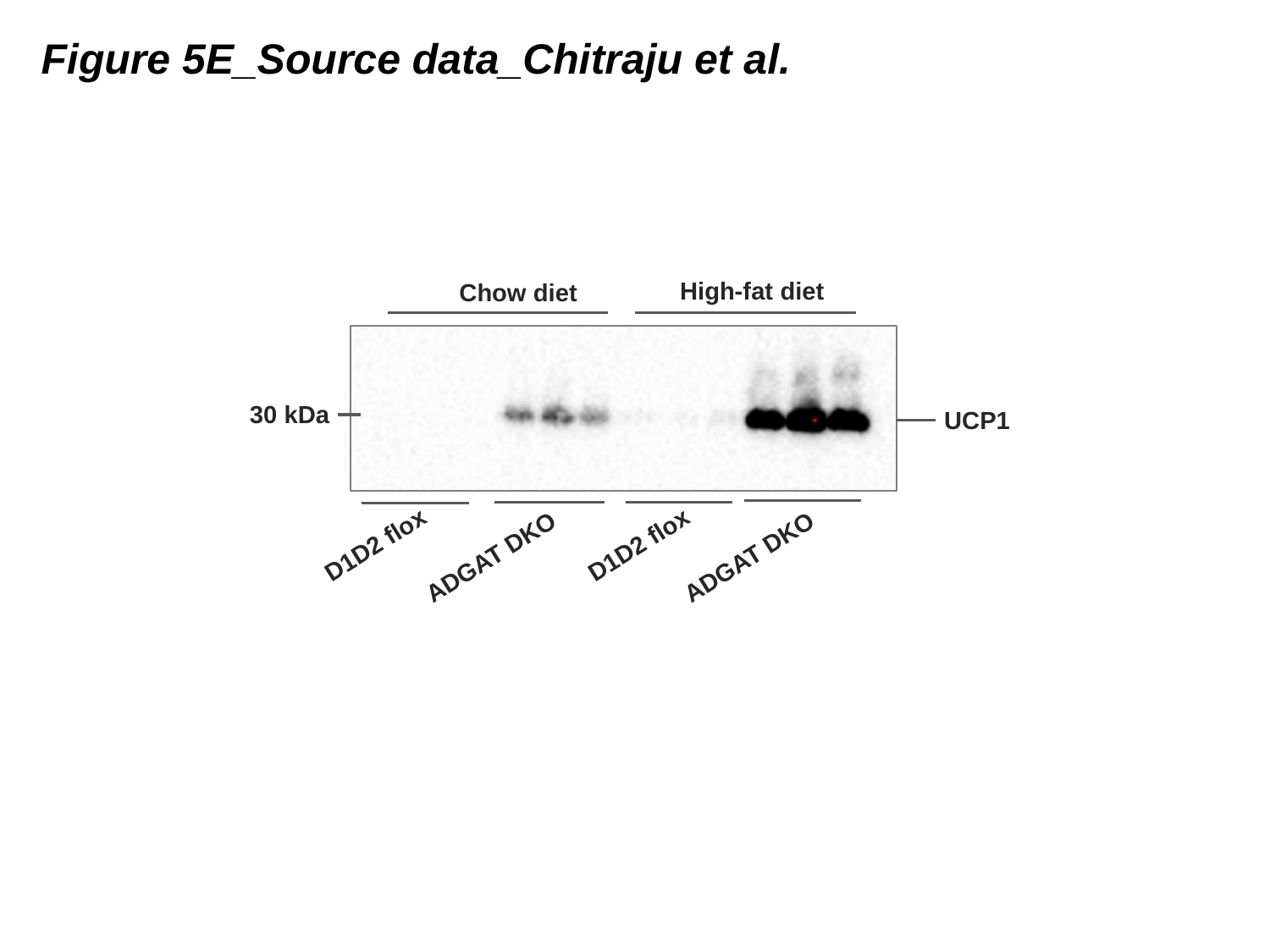

Figure 5E_Source data_Chitraju et al.
High-fat diet
Chow diet
30 kDa
UCP1
D1D2 flox
D1D2 flox
ADGAT DKO
ADGAT DKO

## Slide 2
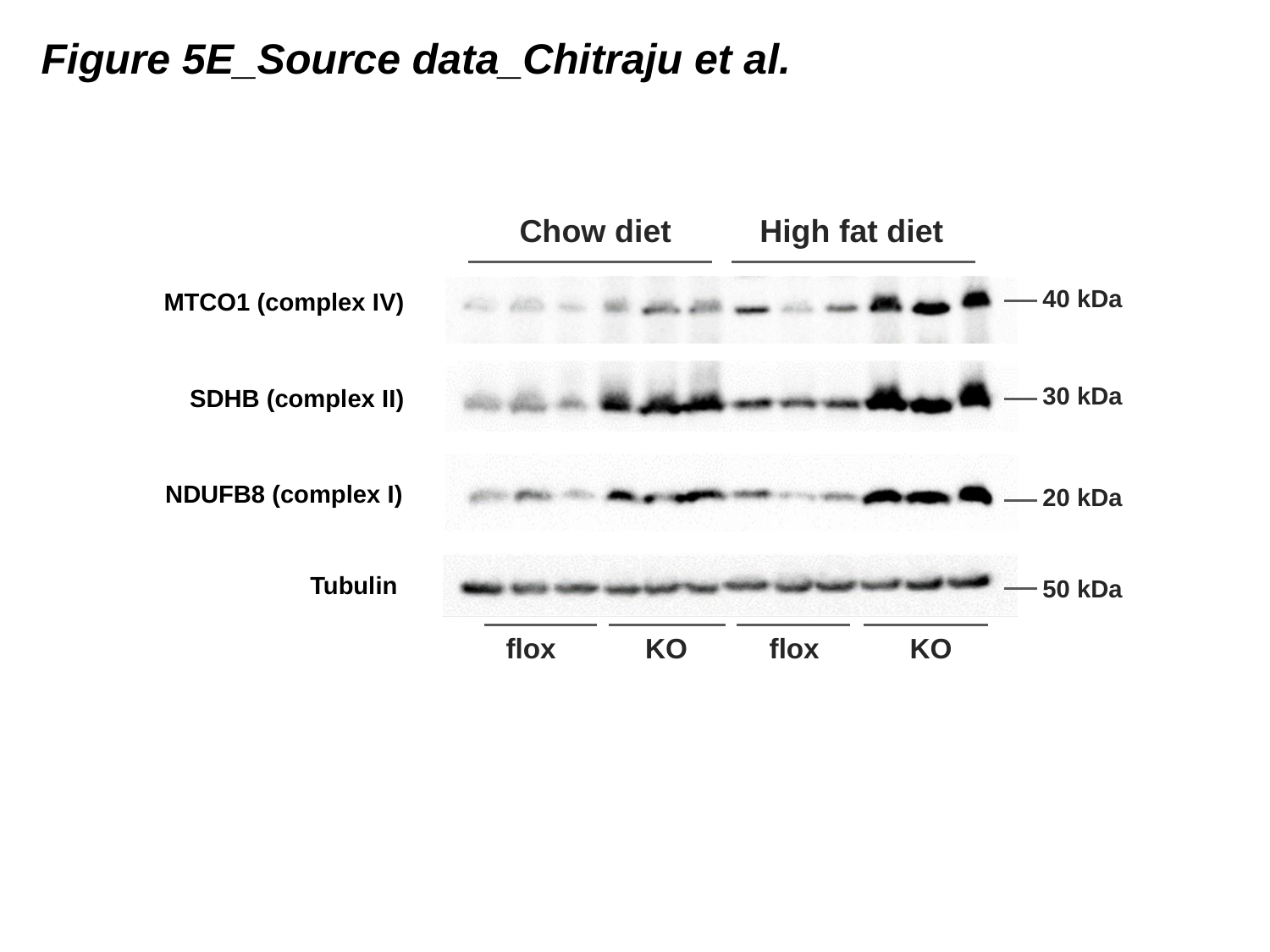

Figure 5E_Source data_Chitraju et al.
Chow diet
High fat diet
40 kDa
MTCO1 (complex IV)
30 kDa
SDHB (complex II)
NDUFB8 (complex I)
20 kDa
Tubulin
50 kDa
flox
KO
flox
KO
